# Charge Neutralization During Peptide Transport in the Bacterial SecYEG Translocon

**DOI:** 10.3390/biom15101442

**Published:** 2025-10-12

**Authors:** Laura Nübl, Ekaterina Sobakinskaya, Frank Müh

**Affiliations:** Department for Theoretical Biophysics, Institute for Theoretical Physics, Johannes Kepler University Linz, Altenberger Strasse 69, 4040 Linz, Austriaesobakinskaya@gmail.com (E.S.)

**Keywords:** confined water, dielectric constant, molecular dynamics simulation, Monte Carlo sampling, p*K_a_* value, Poisson–Boltzmann equation, protonation probability, titration curve, translocation

## Abstract

The driving force behind protein translocation across the cell membrane is not yet fully understood. In bacteria, there is an electrochemical potential across the cell membrane, which can interact with charged residues in the translocation substrate. In this study, the protonation states of lysine and glutamate, serving as test residues in a peptide translocating across the bacterial channel SecYEG, are investigated by applying Poisson–Boltzmann continuum electrostatic free energy calculations and Monte Carlo titrations to snapshots of molecular dynamics (MD) simulations. A clear shift in protonation probability towards the uncharged state is found for both test residues as they move deeper into the channel. Thus, charge neutralization occurs irrespective of whether the original charge of the test residue is positive (lysine) or negative (glutamate). Electrostatic interactions of acidic and basic residues of SecYEG with the peptide cancel out. The main determinants of the test residue’s protonation state are the dielectric properties of its surroundings and interactions with non-titrating charges in the channel. Crucially, the membrane protein—including its water-filled pore—is assigned a low dielectric constant. The results are discussed in the context of the limitations inherent to continuum electrostatics and MD simulations with fixed protonation states.

## 1. Introduction

**Figure 1:**
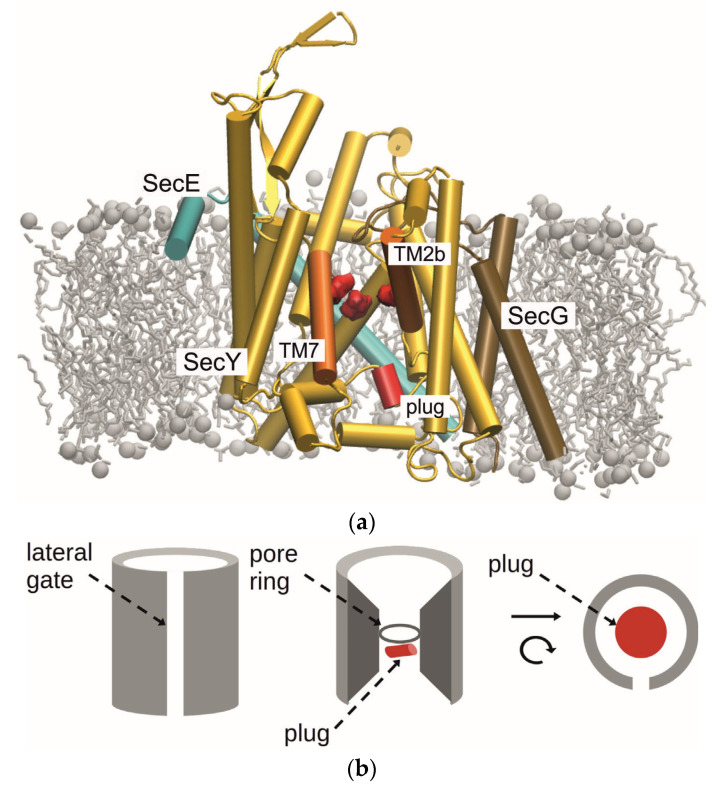
(**a**) Side view of SecYEG embedded in a lipid bilayer in the open state with the cytoplasmic side at the top and the periplasmic side at the bottom. Subunits SecY, SecE, and SecG are shown in yellow, cyan, and brown, respectively, with the exception of the lateral gate helices (TM2b, TM7), which are highlighted in orange. The pore ring (represented by four isoleucine residues) as well as the plug domain are colored red, while the membrane lipids are light gray. Figure made with VMD using the active state version of the *T. thermophilus* channel [5]. (**b**) Cartoon representation of the SecY subunit highlighting its primary features. The hourglass shape of the channel interior and the hydrophobic pore ring can be seen in the center, while the lateral gate is shown on the left and on the right. The importance of the plug domain in sealing the pore is emphasized on the right (Figure adapted from [2]).

In nature, membranes composed of lipid bilayers are used by both pro- and eukaryotic cells to contain their interior structures and protect them from the environment. The tail-to-tail arrangement of the lipids in such membranes gives the structure a hydrophobic core, while the polar head groups point towards the aqueous exterior (see Figure 1a). This provides an efficient barrier to polar and charged (or simply very large) substances on either side, which require the help of channel proteins embedded in the membrane in order to cross [1]. The transportation of proteins or polypeptide chains—which are charged, polar, and very large—is facilitated by specialized channel proteins called translocons.

The Sec (from secretion system) complex is a universally conserved, heterotrimeric membrane protein that is involved in protein transport across, and insertion into, the plasma membrane. In prokaryotes, it is made up of subunits SecY, SecE, and SecG, while the analogous complexes found in eukaryotes and archaea are known as Sec61αβγ and SecYEβ, respectively [2,3,4,5]. The main subunit of SecYEG is SecY, whose ten transmembrane helices (TM) form two halves of a clamshell-like structure. It has a roughly hourglass shape, perpendicular to the membrane plane, with a ring of six hydrophobic amino acids—called the pore ring (PR)—whereby four of the hydrophobic groups define the constriction. For the insertion of proteins into the membrane, the translocon can open its lateral gate (LG), formed by helices TM2b and TM7 [6]. In the resting state, the central pore is blocked by a short helix, TM2a, termed the plug, which moves to the side in the active state [5,7]. Figure 1b shows a cartoon representation of the main features of the channel. Of the other two subunits, only SecE is essential for function by wrapping around the central unit and holding the two halves together. SecG is non-essential for cell viability. The full structure of the heterotrimeric complex is shown in Figure 1a.

In order to activate the channel for translocation or insertion, it must dock to either a ribosome, in what is known as co-translational translocation, or the motor protein SecA for post-translational translocation. In the former, the protein is translocated as it emerges from the ribosome; in the latter, the protein is translated first and is then targeted to the translocon via a signal recognition particle and receptor complex [2]. Once the protein is in the active state, however, the question arises as to what the driving forces are for the subsequent translocation. For the insertion of largely hydrophobic helices into the membrane, it has been shown that the free energy gained by the partitioning into the lipid membrane causes a pulling force on the peptide chain still in the channel [8]. Regarding the translocation through the membrane, extensive research, both experimentally and theoretically, has been performed to uncover the physical mechanisms [2,4,5,9,10]. It appears that there is a kind of consensus on a general ‘‘rough’’ model of activation, which considers a series of steps: (i) association of the channel with the protein to be translocated and ATP-bound SecA, (ii) opening of the LG by the signal sequence (SS) of the transported protein (via the movement of TM7 and TM2b), and (iii) plug relocation. However, details of the physical mechanism underlying activation and operation of the SecY machinery are still poorly understood. Recently, we tried to wrap the available information into a working model based on molecular dynamics (MD) simulations [5]. We came to the conclusion that binding of SecA (in the ATP-bound state) and the SS leads to a transmembrane helix rearrangement that weakens contacts inside the hydrophobic core of SecYEG and provides a driving force for plug opening. The conformational transitions are enabled by a delicate interplay between hydrophobic forces on one side, and a combination of forces on the other side that we termed PEES. This abbreviation stands for the proton motive force, external forces (due to binding with the translocation partners), entropic effects (e.g., due to conformational changes in the protein), and solvent-induced forces (e.g., due to bound water). In the open state, SecYEG still provides a barrier for bulky residues, meaning translocation is very likely not just a diffusion process, but pulling forces are required. In this context, it has been hypothesized earlier that the proton motive force plays a significant role in translocation [10].

The proton motive force (PMF) is defined as the sum of the transmembrane potential and the transmembrane proton gradient, and it is an essential source of energy for cellular processes [11]. In *Escherichia coli* cells, the transmembrane potential is about 100–150 mV (negative inside), and has been shown to influence the translocation of model peptides containing presumably negatively charged residues [12]. However, as proteins can contain both acidic and basic amino acids—and generally have similar amounts of both—this means that, given the $pK_{a}$ values of those residues at physiological pH values, it is questionable that there is a net force supporting translocation [10]. Since $pK_{a}$ values of titratable groups depend on the electrostatic environment in which they are located, it is possible that shifts in $pK_{a}$ values may cause a net charge of the substrate due to different charge states in acids and bases [10]. If the electrostatic interactions in the translocon stabilize negative charges, to compensate for them existing in an area of lower permittivity, while not doing the same for positively charged residues, there would be a net force acting in the direction of translocation.

**Figure 2:**
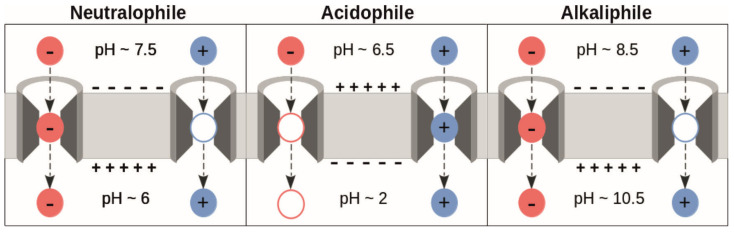
Summary of charge states of acidic (red) and basic (blue) residues over the course of translocation that would allow a net force to act in the direction of translocation; empty outlines imply the uncharged state of the residues. The black, dashed arrows show the direction of translocation (cytoplasm to periplasm), while the plus and minus symbols on either side of the membrane represent the transmembrane potential. The values are taken from [10], and the figure is an adapted version of the one published therein.

Additionally, while the above considerations are based on the transmembrane potential and pH values of neutralophilic bacteria, the mechanism could still work in the direction of translocation in extremophilic bacteria [10], if the $pK_{a}$ shifts experienced by acids and bases lead to the protonation states shown in Figure 2. Additionally, the electrostatic interactions with titratable residues in SecYEG could stabilize one charge over another in the different bacteria, as the specific protonation state of the translocon is pH dependent, too. However, there must be an alternative driving force involved in protein translocation in eukaryotic cells, as Sec61 is located in the endoplasmic reticulum (ER) membrane which does not have an appreciable transmembrane potential [10,13].

The goal of the present work is to computationally determine the titration behavior of amino acids during their translocation across SecYEG in order to gain insight into the driving forces behind this process.

## 2. Theory and Methods

### 2.1. Electrostatics

Different kinds of methods have been devised to assess the $pK_{a}$ values of titratable groups in proteins, as well as the proteins’ protonation states [14,15]. Among the physics-based computational methods, approaches combining Poisson–Boltzmann (PB) electrostatics with Monte Carlo (MC) sampling of protonation states are popular due to their cost–benefit ratio [16,17,18,19]. In the PB approach, proteins are modeled as a set of atomic partial charges (APCs) embedded in a dielectric continuum. The APCs are placed on atomic positions, which in the present work are derived from molecular dynamics (MD) simulations. The method is based on the Poisson equation:

(1)
∇
·
ε
(
r
)
∇Φ
r
=
−
ρ
r
ε
0
.

Here, $\varepsilon_{0}$ is the vacuum permittivity. The APCs $q_{i}$ placed at the atomic positions $r_{i}$ are represented by the charge density:

(2) ρ
r=∑iqiδ(
r−
ri).

The electrostatic potential $\Phi \left(r\right)$ is obtained from a numerical solution of the differential Equation (1) and used to compute interaction energies of titratable groups as detailed below and in the literature [16,17,18,20]. The relative dielectric permittivity ε(r) describes the continuum in which the APCs are embedded as a heterogeneous dielectric medium with a piecewise constant electric polarizability. A distinction is made between the protein interior (ε
r = εprotein), the lipid bilayer (ε
r = εmem), and the surrounding aqueous solution ($\varepsilon (r)\;=\;\varepsilon_{aq}$).

For the description of proteins in a realistic environment, one has to take into account the effects of mobile ions in the aqueous phase. This is done by considering the total charge density in Equation (1) instead of ρ
r:

(3) ρtotal
r=ρ
r+ρion
r,

where

(4) ρion
r=NA∑scs
rqse−βqsΦ(
r)

represents the mobile ions. Here, β is the inverse thermal energy $k_{B}T$ (with $k_{B}$ being Boltzmann’s constant and T the absolute temperature) and $N_{A}$ is Avogadro’s constant, while $c_{s}$ and $q_{s}$ are, respectively, the molar concentration and charge of ion species s. Equation (1) with ρtotal
r at the right-hand side (rhs) is referred to as the Poisson–Boltzmann equation (PBE). For small potentials ( $q_{s}\Phi \;\approx \;k_{B}T$), and preferably monovalent ions, the exponential term can be simplified by doing a Taylor expansion to the first order:

(5) ∑scs
rqse−βqsΦ(
r)≈∑scs
rqs−βcs
rqs2Φ(r).

In Equation (5), the first term on the rhs is equal to zero due to the condition of electroneutrality. To further simplify the remaining term, the ionic strength I(
r) is introduced according to

(6) $$I\left(r\right)=\frac{1}{2}\sum_{s}c_{s}\left(r\right)z_{s}^{2},$$

where $z_{s}=q_{s}/e$ is the charge number of ion type s, and e is the elementary charge. Note that $I\left(r\right)$ depends on position, since it is nonzero only in the aqueous phase outside the protein and the membrane. Finally, by substituting the simplified terms into the PBE, the linearized Poisson–Boltzmann equation (LPBE) is obtained:

(7) $$\varepsilon_{0}\nabla \cdot \left[\varepsilon \left(r\right)\nabla \Phi \left(r\right)\right]=-\rho \left(r\right)+2N_{A}\beta e^{2}I\left(r\right)\Phi \left(r\right).$$

### 2.2. Protonation Patterns

**Figure 3:**
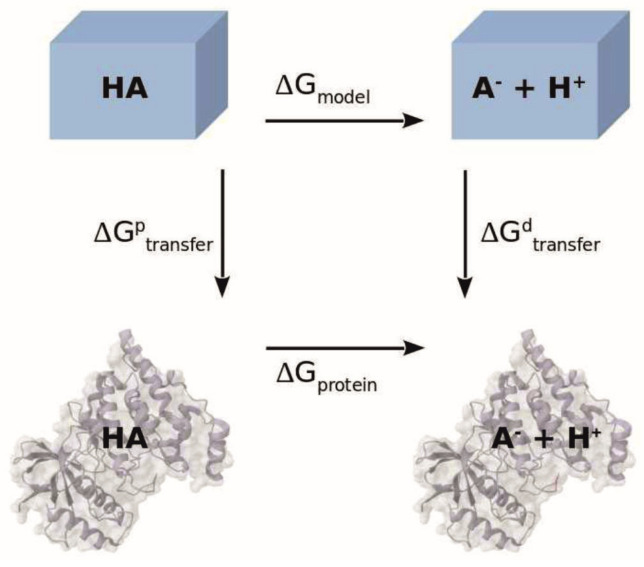
Thermodynamic cycle used to determine the free energy of deprotonation of a titratable group A in a protein environment. The blue cubes represent the aqueous environment in which the model compound titrations occur, while the molecular surface and cartoon structure in gray represent the group being in a protein environment.

The calculation of protonation states in proteins with multiple titratable residues can be done by determining the electrostatic work necessary to transfer each titratable residue in the protonated and deprotonated state from an aqueous medium into the protein environment, as well as computing the interaction energies with other titratable groups. This type of computation is based on the thermodynamic cycle shown in Figure 3, from which the free energy of deprotonation of a titratable group in the protein, $∆G_{protein}$, is obtained as [18,20,21]:

(8) $$∆G_{protein}=∆G_{model}+∆G_{transfer}^{d}-∆G_{transfer}^{p}.$$

The free energy of deprotonation of the model compound, $∆G_{model}$, is inferred from experimental $pK_{a}$ data [17]. Electrostatic computations are used to determine the free energy of transfer of the group A from an aqueous to a protein environment ( $∆G_{transfer}$ with superscripts p and d for the protonated and deprotonated states, respectively).

The primary assumption of such a model is that the $pK_{a}$ of an ionizing group in the protein is that of a model compound (the same amino acid but isolated and with capping groups placed on its termini), shifted solely due to electrostatic interactions with the group’s surroundings; any conformational changes and steric effects are neglected [22]. In the present work, we do not employ a single structure, but structural ensembles generated by MD simulations. The second assumption is that only a subset of APCs in the group change when the protonation state is changed (referred to as the titrating charges, $Q_{\alpha }$), while the remaining APCs are constant (non-titrating charges, $q_{i}$). When only one ionizable group is considered, meaning all other residues are in their uncharged state, the $pK_{a}$ is the so-called intrinsic $pK_{a}$ [20,23], $pK_{a,intr}$:

(9) $$pK_{a,intr}=pK_{a,model}+\frac{∆G_{transfer}^{d}-∆G_{transfer}^{p}}{RT\ln10}\;=pK_{a,model}+\frac{\left(W_{protein}^{p}-W_{protein}^{d}\right)-\left(W_{model}^{p}-W_{model}^{d}\right)}{RT\ln10}.$$

Here, energies are given per mole—as indicated by the use of the gas constant R. The ∆W terms, then, are the work required to create the charge distributions of those states. Writing the explicit contributions to each W term, the interaction between non-titrating charges in the protein will cancel between the $W^{p}$ and $W^{d}$ terms as the $q_{i}$ do not change during a protonation state change. The Coulomb interactions of titrating charges will cancel between the $W_{model}$ and $W_{protein}$ expressions for each state, as they are identical in the protein and model compounds. This leaves only the interactions of the titrating and non-titrating charges with the electrostatic potential (ESP), which are called the Born and background terms by convention. Therefore, the $pK_{a,intr}$ can be re-written as

(10) $$pK_{a,intr}=pK_{a,model}+\frac{\Delta ∆G_{Born}+\Delta ∆G_{bg}}{RT\ln10}.$$

The Born term, $\Delta ∆G_{Born}$, results from the interaction of the titrating charges $Q_{\alpha }$ with the potentials $\Phi_{protein}^{p}$, $\Phi_{protein}^{d}$ of the protein and $\Phi_{model}^{p}$, $\Phi_{model}^{d}$ of the model compound—determined by solving the LPBE (Equation (7)) with the charge distribution $\rho \left(r\right)$ comprising only the titrating charges $Q_{\alpha }^{p}$ or $Q_{\alpha }^{d}$, and with the spatial dependence of $\varepsilon \left(r\right)$ and $I\left(r\right)$ being determined by the molecular surfaces of the protein and the model compound:

(11) $$\Delta ∆G_{Born}=\frac{1}{2}\sum_{\alpha }\left\{Q_{\alpha }^{p}\left[\Phi_{protein}^{p}\left(r_{\alpha }\right)-\Phi_{model}^{p}\left(r_{\alpha }\right)\right]\right.\;\left.-Q_{\alpha }^{d}\left[\Phi_{protein}^{d}\left(r_{\alpha }\right)-\Phi_{model}^{d}\left(r_{\alpha }\right)\right]\right\}.$$

The background (bg) term represents the interaction of the non-titrating (background) charges $q_{i}$ with the various potentials:

(12) $$\Delta ∆G_{bg}=\sum_{i}q_{i,protein}\left[\Phi_{protein}^{p}\left(r_{i}\right)-\Phi_{protein}^{d}\left(r_{i}\right)\right]\;-\sum_{k}q_{k,model}\left[\Phi_{model}^{p}\left(r_{k}\right)-\Phi_{model}^{d}\left(r_{k}\right)\right].$$

This leads to the LPBE being solved four times for each titratable site.

A protein with N titratable sites, having two different protonation states each (d or p), has a total of $2^{N}$ protonation patterns, which can be described by an N-dimensional vector $x_{\sigma }=\left(x_{1}^{\sigma },\;x_{2}^{\sigma },\ldots ,x_{N}^{\sigma }\right)$ of binary elements representing the charge state of each site ( $x_{i}^{\sigma }=0$ for deprotonated, $x_{i}^{\sigma }=1$ for protonated; i=1,…,N). In the following, we shall use the term “pattern” for such multisite states—to better distinguish them from the protonation states of individual sites—and denote the protein in such a multisite state by $M\left(x_{\sigma }\right)$. The index $\sigma =1,\ldots ,2^{N}$ counts the various patterns. Let the vector 0 represent the reference pattern, in which all sites are in their neutral state. The entries in this vector are $x_{i}^{0}=1$ for acids and $x_{i}^{0}=0$ for bases. Following [16], we consider the chemical equilibrium between an arbitrary pattern $x_{\sigma }$ and the reference pattern:

(13) $$M\left(0\right)+\nu \left(x_{\sigma }\right)H^{+}⇌M\left(x_{\sigma }\right).$$

Here, $\nu \left(x_{\sigma }\right)$ is the net number of protons that would be released on going from pattern $x_{\sigma }$ to the reference pattern. Note that $\nu \left(x_{\sigma }\right)$ may be negative, which would correspond to the situation that a net proton uptake leads from $M\left(x_{\sigma }\right)$ to $M\left(0\right)$. The net proton number can be written as a sum over sites:

(14) $$\nu \left(x_{\sigma }\right)=\sum_{i}\nu_{i}(x_{i}^{\sigma }),$$

where $\nu_{i}\left(x_{i}^{\sigma }\right)=x_{i}^{\sigma }-x_{i}^{0}\;\in \;\left\{-1,0,+1\right\}$. Then, the free energy change for going from $M\left(0\right)$ to $M\left(x\right)$ at some fixed pH is [16]:

(15) $$\Delta G\left(x_{\sigma },pH\right)=\sum_{i}\nu_{i}\left(x_{i}^{\sigma }\right)2.303\;RT\left(pH-pK_{i,intr}\right)+\frac{1}{2}\sum_{\begin{array}{c} ij\\ i\;\neq j \end{array}}^{N}W_{ij}\nu_{i}\left(x_{i}^{\sigma }\right)\nu_{j}\left(x_{j}^{\sigma }\right).$$

The first sum contains the changes in free energy due to the acid dissociation reactions of individual sites i, while the second term sums up all additional work originating from pairs of sites being in their non-reference states. Note that the entries in the double sum are non-zero only if both sites i and j are charged. The interaction energy is given by

(16) $$W_{ij}=\sum_{\alpha }^{site\;i}(Q_{\alpha }^{p}-Q_{\alpha }^{d})\left[\Phi_{j}^{p}\left(r_{\alpha }\right)-\Phi_{j}^{d}\left(r_{\alpha }\right)\right],$$

with $Q_{\alpha }$ being the titrating charges of site i, and $\Phi_{j}$ the potentials due to the titrating charges of site j originating from the solution of the LPBE.

Using the free energy change, the protonation probability $x_{i}$ of the individual site i as a function of pH can be expressed as a thermodynamic average over all protonation patterns of the protein [17]:

(17) $$\langle x_{i}\rangle =\frac{\sum_{\sigma =1}^{2^{N}}x_{i}^{\sigma }\exp\left(\Delta G\left(x_{\sigma },pH\right)/RT\right)}{\sum_{\sigma =1}^{2^{N}}\exp\left(\Delta G\left(x_{\sigma },pH\right)/RT\right)}.$$

Note that $x_{i}$ can be any number between 0 and 1 in contrast to the binary variable $x_{i}^{\sigma }$. The protonation probabilities can be plotted against the pH to yield titration curves. In general, the number $2^{N}$ is too large for a direct computation of the protonation probabilities on the basis of Equation (17). The solution to this problem is the use of a MC technique (see below).

### 2.3. Computational Methods

#### 2.3.1. Preparation of Structures and General Setup of Molecular Dynamics Simulations

In order to determine the ($pK_{a}$ changes experienced by amino acids in a translocating peptide—as a result of their position in the translocon SecYEG—constant velocity steered molecular dynamics (SMD) simulations were performed. The peptide was pulled through the channel and the titration behavior of residue 31 was investigated.

**Figure 4:**
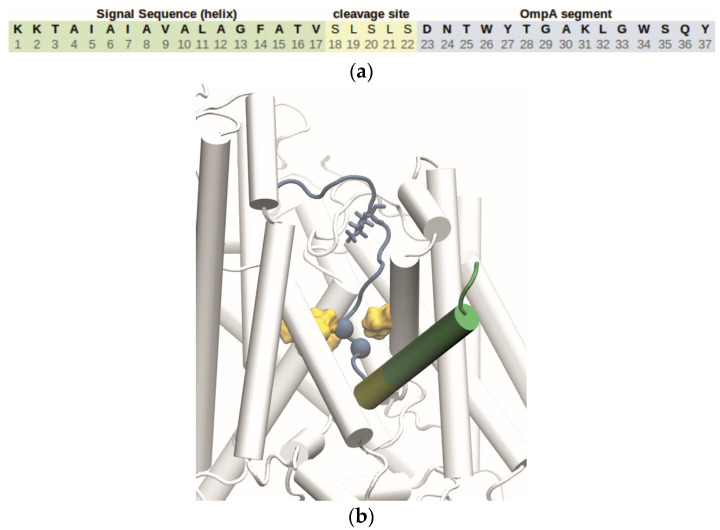
(**a**) Primary sequence of the pre-protein construct (referred to as “peptide”) used in the simulations including the signal sequence (SS), cleavage site, and the actual translocation substrate (OmpA segment). Position 31, which is a lysine in this sequence, marks the residue used to test the titration behavior. Positions 24 and 25 (C_α_-atoms) were tagged as SMD atoms. (**b**) Visualization of peptide structure at the start of the SMD simulations, with color coding for the different sections as described in (**a**). The blue beads correspond to the SMD atoms, while the lysine shown in licorice representation is the test residue at position 31. The position of the PR is indicated by four Ile residues shown in yellow. In the shown snapshot, the C_α_-atom of residue 25 is located in the PR.

For the active state model of the Sec channel, the resting-state crystal structure of the *Thermus thermophilus* channel (PDB ID 5AWW [24]) was opened by moving its main helices and plug domain based on the coordinates of an active-state chimera structure (*Geobacillus thermodenitrificans* SecYE and *Bacillus subtilis* SecA; PDB ID 5EUL [25]) as described in our previous work [5,26]. The peptide used is composed of the SS, cleavage site, and first strand of the outer membrane protein A (OmpA) β-barrel, whose structure was created from its primary sequence (Figure 4) before being manually placed inside the channel using a CHARMM script [27]. These steps were part of a previous project (see [5] for details). In our simulations, we only consider the OmpA segment (Figure 4a) to be translocated, while the SS is bound to the LG (Figure 4b) and the cleavage site including Asp 23 is located below the PR. Thus, Lys 31 is the only charged residue in the actually translocating peptide segment and, therefore, was chosen as the test residue.

MD simulations were performed with NAMD version 2.13 [28] along with the CHARMM36m force field [29,30]. The TIP3P model was used for water molecules [31]. All simulations were run under (NPT conditions: constant temperature control was regulated with Langevin dynamics [32] with a 5 ps^−1^ damping coefficient coupled to all heavy atoms ((T = 300 K); constant pressure was maintained at 1 atm by a Nosé–Hoover Langevin piston barostat [33,34] with decay period 100 fs and a damping time of 50 fs. Electrostatic interactions were computed with particle-mesh Ewald summation using a grid spacing of 1 Å and a cut-off of the short-range real-space interactions at 12 Å with a smooth switch function beginning at 10 Å. The equations of motion were integrated in 2 fs or 1 fs steps using the velocity Verlet algorithm. The number of time steps between evaluations of the short-range non-bonded interactions and the full electrostatic interactions were equal to 2 and 4, respectively. The SHAKE algorithm was employed to constrain the length of the bonds involving hydrogen atoms. Coordinates were saved every 2 ps.

In our previous work [5], steered MD (SMD) with a constant velocity protocol [35] implemented in NAMD was used to pull the peptide through SecYEG in order to define various positions for umbrella sampling. Here, we used the same technique to define different positions of residue 31 of the peptide inside the channel for sampling of protonation states. The present SMD was performed with a step size of 1 fs. Harmonic constraints with a force constant of 1 kcal/(mol Å^2^) were placed on the protein backbone atoms as well as the plug domain and a linker segment. To pull the peptide, the C_α_-atom of residue 24 of the peptide was tagged as the SMD atom, which was then pulled through the pore along the (z-axis (set perpendicular to the membrane, see [5,26]) with a force constant of 36 kcal/(mol Å^2^) at a velocity of 2 Å/ns. The simulation was run for 10 ns, followed by four additional runs for 7 ns each. After run 2, pulling was switched to the C_α_-atom of residue 25 of the peptide to avoid the unwinding of the signal sequence helix. Snapshots of the system were then taken from the trajectories at regular intervals, covering various positions of residue 31 in the channel. In the following, we will refer to these positions as “frames”.

#### 2.3.2. Preparation and Titration with Residue 31 Being Lysine

**Figure 5:**
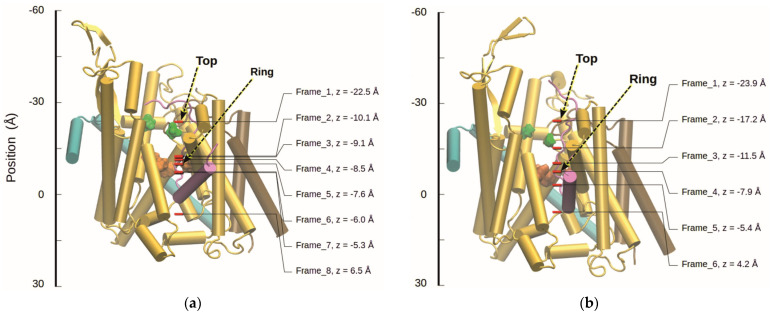
Visualizations of SecYEG with a translocating peptide in the cartoon representation. The protein subunits SecY, E, and G are shown in gold, teal, and brown, respectively, while the peptide and signal sequence are colored magenta. Asp Y410 on SecY and Asp G34 on SecG are displayed in green, and the pore ring is represented by the Ile residues Y81, Y188, Y275, and Y403 on SecY in orange. The red bars correspond to the positions of peptide residue 31 in the frames chosen for titration and are labeled with the frame number and the position of the C_α_-atom on the (z-axis. In the case of Lys 31 (**a**), frames 1 and 5 correspond to the “top” and “ring” frames used for simulations with altered protonation forms. In the case of Glu 31 (**b**), frames 1 and 4 were chosen. Figures made with VMD [36].

Initial titrations were done on snapshots of the active-state structure of SecYEG with the peptide containing Lys 31 taken from our previous work [5]. A total of 8 snapshots in different stages of translocation—corresponding to 8 frames (see Figure 5a)—were used as starting conformations for 10 ns MD simulations. The simulation parameters were kept the same as for the SMD simulation except that the constraints were modified. Harmonic constraints were placed on the C_α_-atom of peptide residue 31 as well as the backbone of the plug domain and helices 5, 7, 8, and 9 of SecY. Force constants of 0.4 and 1 kcal/(mol Å^2^) were used, respectively. From these trajectories, 50 structures were taken from the final 5 ns (assuming that these represent a thermally equilibrated ensemble) and the structure files edited for titration by adding uncharged water and dummy atoms to the pore (see Section 2.3.4). Titrations were then performed on these structures as described in Section 2.3.4, Section 2.3.5, Section 2.3.6.

#### 2.3.3. Preparation and Titration with Residue 31 Being Glutamic Acid

In order to determine the ($pK_{a}$ shifts experienced by a negatively charged residue, Lys 31 in the peptide was changed to Glu using CHARMM [27], before it was placed into the active state structure of the SecYEG translocon. Once the residue was mutated, energy minimization was performed on the system according to the following protocol: (1) water only: all atoms except water were fixed; (2) membrane and water: protein atoms were fixed; (3) protein, membrane, and water: all N, CA, C, and O atoms were fixed; (4) all: no atoms were fixed. In each step, 500 steps of minimization using the steepest descent (SD, TOLG 0.1) and adopted basis Newton-Raphson (ABNR, TOLG 0.01) algorithms were performed, except for the final ABNR minimization, which was set to 1000 steps. The non-bonded list was regenerated every 20 steps.

Following the energy-minimization, the system was equilibrated with NAMD [28] in six consecutive steps with decreasing constraints with each run. The simulation parameters are as follows: (1) 1 ns of simulation time, with force constants of 5 and 0.5 kcal/(mol Å^2^) on the protein backbone and membrane atoms, respectively; (2) 1 ns of simulation time with the force constants lowered to 2 and 0.2 kcal/(mol Å^2^); (3) 1 ns of simulation time with the force constant lowered to 1 kcal/(mol Å^2^) for the protein, and everything else is released. For the final three steps, more specialized constraints were placed on the following selections: (i) SecY helices 5, 7, 8, 10, parts of helix 9, and the backbone of SecE, (ii) KCl ions, (iii) the plug domain, (iv) the translocating peptide: (4) 1 ns of simulation time with force constants of 1, 0.2, 1, 0.2 kcal/(mol Å^2^); (5) 1 ns of simulation time with force constants of 1, 0.1, 1, 0.1 kcal/(mol Å^2^); (6) 5 ns of simulation time with only the constraints on SecYE in place at 1 kcal/(mol Å^2^). The constraints could not be removed completely as the system is based on the modified crystal structure of a closed channel—without a docked ribosome or SecA—and so SecY would have closed again if left unconstrained (see [5]).

Once the system had been equilibrated for a total of 10 ns simulation time, the final structure was taken from the trajectory and used as the starting conformation of the constant-velocity SMD simulation for the translocation of the peptide. The parameters used for SMD were the same as described for the Lys-peptide in Section 2.3.2. Snapshots of the system were then taken from the trajectory files at regular intervals. Before titrations were performed on these frames (see Figure 5b), the snapshots were used as starting conformations for 10 ns trajectories as for the Lys-case. From these trajectories, 50 structures were taken from the final 5 ns, edited by adding uncharged water and dummy atoms, and titrated as described below.

#### 2.3.4. Poisson–Boltzmann Computations

**Figure 6:**
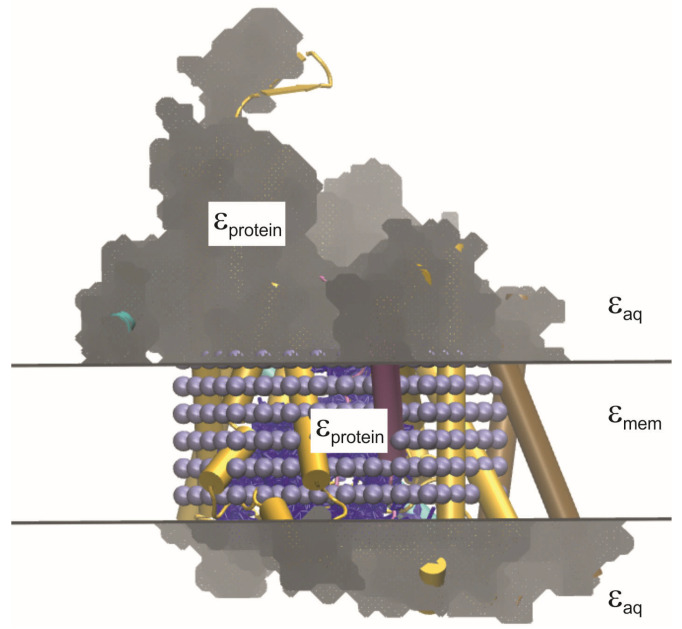
Visualization of the solvent-excluded surface calculated for SecYEG with uncharged dummy atoms (ice blue spheres) and water molecules (dark blue lines) placed in the channel. The gray mesh shows the interface between ($\varepsilon_{aq}$ and ($\varepsilon_{protein}$, and the horizontal lines the interface between ($\varepsilon_{aq}$ and the membrane slab (($\varepsilon_{mem}=2.0$).

PB computations based on the theory described in Section 2.1 and Section 2.2 were performed with the solver TABPS developed by Rabenstein and Kieseritzky in the group of Knapp [37,38]. TAPBS is a front-end connected to the Adaptive Poisson–Boltzmann Solver APBS [39]. The LPBE is solved by a finite-difference technique [40] employing a grid-focusing algorithm with cubic grids. The grid spacings were 3, 1, and 0.25 Å for the titratable group in the protein environment (three grids) as well as 1 and 0.25 Å for the model compound (two grids). The ionic strength was set to that of a 0.1 M NaCl solution with an ion radius of 2 Å defining the ion exclusion layer. The solvent radius defining the boundaries between different values of ($\varepsilon \left(r\right)$ (see Section 2.1.) via the solvent-excluded surface (SES) [41,42,43] was set to 1.4 Å as usually assumed for water. For the PB computations, all lipids constituting the membrane, water molecules, and explicit ions in the aqueous phase were removed, and the protein part was placed in a membrane slab with ($\varepsilon_{mem}=2.0$ as illustrated in Figure 6. We used ($\varepsilon_{aq}=80$ and ($\varepsilon_{protein}=4$. The latter value is a compromise for the protein, which is actually heterogeneous, and lies in a range suggested by MD simulations (ε=3−6) [44], theoretical estimates (ε=2.5−4) [45] as well as a recent Kramers-Kronig analysis of the spectroscopic properties of bovine serum albumin (BSA; dry powder, (ε=3.6−3.7) [46].

Based on test titrations, it was found that the surface calculations to obtain ($\varepsilon \left(r\right)$ resulted in “bubble artifacts” of high dielectric permittivity inside the pore. This is due to an in-build feature of TAPBS [37] that automatically assigns ($\varepsilon_{aq}$ to any region inside the protein structure (cavity) able to fit a sphere of radius 1.4 Å. Therefore, in order to keep the dielectric constant inside the transmembrane parts of the protein homogeneous, the structure files were edited to place uncharged atoms inside the pore and block the probe from entering. For this purpose, scripts were written using the python library MDAnalysis [47,48]: From each frame, 50 structures were extracted from the final 5 ns, and water molecules in the channel were selected based on their coordinates. PDB files were created for each structure consisting of the protein coordinates, the coordinates of the selected channel waters from the first 20 time steps, as well as the coordinates of dummy atoms (with a radius of 2 Å) placed at even intervals throughout the trans-membrane region of SecY (Figure 6). The charges of both water and dummy atoms were set to zero (cf. Supplementary Material SM1).

#### 2.3.5. Protonation Forms

**Figure 7:**
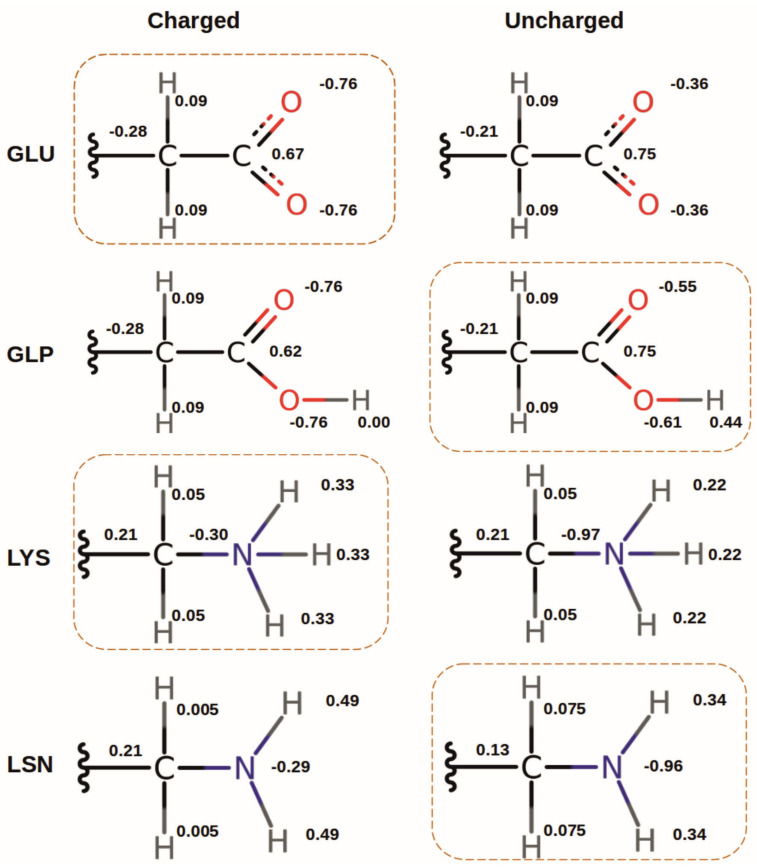
APCs assigned to side chain atoms of Glu and Lys in two different protonation forms. GLU and LYS are the standard forms for simulations (at physiological pH), as they correspond to the structures of the amino acids when charged, which is the most likely state based on their standard ($pK_{a}$ values. For the neutral forms, GLU must be protonated (⟹ GLP) and LYS deprotonated (⟹ LSN). For each protonation form, the APCs are assigned to reflect the charged and uncharged state of the residue for titration calculations. Forms with dashed framing correspond to those used in the MD simulations. Note that in these cases, protonation form and protonation state coincide.

PB-based approaches to protonation states in proteins, using a purely electrostatic, continuum dielectric model as described in Section 2.1, were primarily designed to predict ($pK_{a}$ values based on X-ray structures [22,23,49,50]. Since hydrogen atoms could not (and nowadays often still cannot) be resolved in X-ray structures of macromolecules, earlier calculations modeled the protonation/deprotonation by changing APCs on heavy atoms of the titrating site [22,23]. Later refinements employed explicitly represented protons [51] with proton positions inferred from modeling (e.g., with CHARMM [27]). In these models, the number of atoms in the deprotonated form is still the same as in the protonated form, i.e., deprotonation is realized implicitly by changing APCs (see Figure 7). In the present work, all atom positions are inferred from MD simulations, so that all protons are—in principle—explicitly modeled. However, in MD simulations a standard protonation state of each titratable site is usually assumed, implying that acids are deprotonated and negatively charged, and bases are protonated and positively charged. It is possible that these fixed protonation states bias the dynamics because of both the fixed APCs and the prescribed absence or presence of explicit protons.

In order to investigate this bias, we performed variants of the simulations with residue 31 of the peptide in different *protonation forms*. By protonation “form” we mean the explicit absence or presence of a hydrogen atom in the structure (Figure 7). In the following, the protonation forms will be indicated by a three-letter code with capital letters, while the amino acids, in general, will be referred to by a three-letter code without capitalization. For example, Glu can be in the protonation form GLU with no explicit proton at the carboxyl group, and in the form GLP with one explicit proton. Likewise, Lys can be in the protonation form LYS with three protons at the amino group, or in the form LSN with only two protons. The non-standard forms GLP and LSN were created by employing the CHARMM patches GLUP and LSN, respectively (for APCs, see Supplementary Material SM1). Note that each protonation form can still exist in two protonation states determined by the APCs as illustrated in Figure 7. In this way, each form used in the MD simulation can be subjected to a PB-based determination of the protonation probability. As the residue state description (st) files used by TAPBS to calculate the intrinsic ($pK_{a}$ values and interactions of titratable residues do not include LSN or GLP forms, new files were created manually based on LYS and GLU state description files (see Supplementary Materials SM2 and SM3).

Histidine is a special case, as its side chain is usually represented by three protonation states (including two uncharged tautomers [52]) with the corresponding protonation forms HSD, HSE, and HSP in CHARMM. Both protons must be included explicitly (the HSP form) in order to apply the standard titration protocol. The charged version would have to be used for the MD simulations, which would affect the dynamics. Determining the magnitude of this effect would require three separate MD simulations to be done for each of the protonation forms, which was considered too computationally expensive. Since there are only two His residues in the translocon and neither one is in the top half of the pore, we neglected His as a titratable residue in the present work.

#### 2.3.6. Monte-Carlo Titration of Protonation Patterns

The MC titrations were performed with the program Karlsberg2 developed by Rabenstein and Kieseritzky in the Knapp group [37,38]. This program uses the intrinsic ($pK_{a}$ values of each titratable site and the corresponding matrix ($W_{ij}$ of charged-site interactions produced by TAPBS (cf. Section 2.3.4). Titrations were performed from pH 0 to 14 in increments of 0.5 pH units at 300 K. The minimum absolute interaction energy to consider double moves for a strongly coupled pair [17,53] was set to 2.5 pH units, and 5 pH units for triple moves. The number of scans was set to ($10^{5}$ for MC scans with all sites, as well as for scans with the reduced set of sites, which was determined by a reduced set tolerance of ($10^{-4}$. The correlation limit was set to 0.1, and the maximum correlation time to 100 scans.

## 3. Results

Following an SMD simulation of the translocation, the $pK_{a}$ shift in peptide residue 31 as a result of its position was investigated by performing continuum electrostatics calculations and MC titrations with the residue at selected positions in the channel. For this purpose, snapshots were taken from the SMD trajectory and used as starting structures for MD simulations with positional constraints on the C_α_-atom of the residue. The positions chosen can be seen in Figure 5.

**Figure 8:**
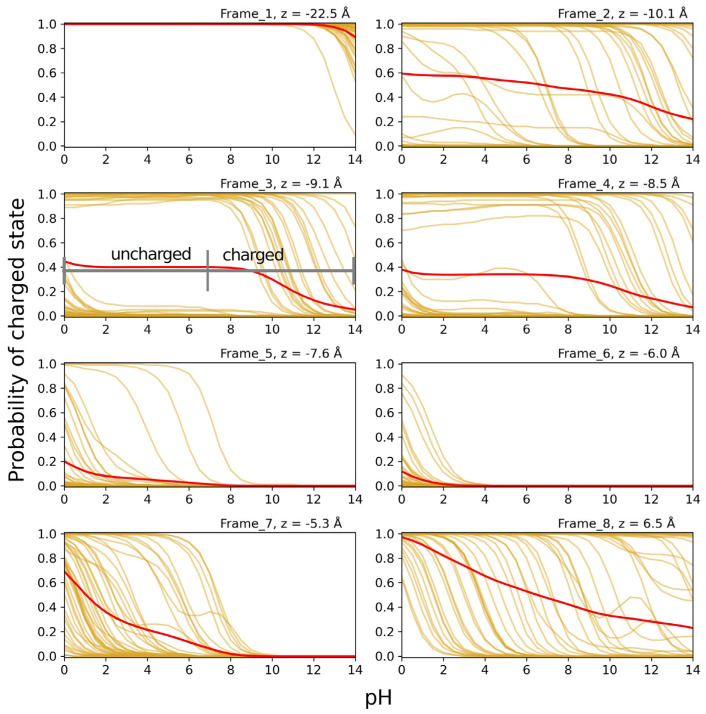
Titration curves of peptide residue LYS 31 over the course of translocation. The curves in frames 1–8 are each based on 50 structures from constrained MD simulations with the position of residue 31 determined by constant velocity SMD simulations pulling the peptide through the channel. The frames correspond to the positions indicated in Figure 5a. The golden curves are individual titration curves of the 50 structures, while the red lines correspond to the average of the 50 structures in each frame to illustrate the average protonation probability (=probability of being positively charged). The “charged” and “uncharged” labels on frame 3 highlight the binary behavior in that frame, which can also be seen in frame 4, as well as, to a lesser extent, in frame 2.

In Figure 8, the titration curves for Lys at position 31 for structures taken from MD simulations with Lys 31 in the LYS protonation form (cf. Figure 7) are shown for the eight different positions in the SecYEG channel. Titrations were performed on 50 structures for each frame. A clear trend can be seen in the average probability of the charged state decreasing as the residue moves farther into the channel (frames 1 to 5), and increasing again once it has passed the PR (frames 6 to 8). The form of some individual titration curves deviates strongly from the standard Henderson-Hasselbalch (HH) form, indicating strong coupling of Lys 31 to other titratable groups above and below the PR. The titration curves for frames 3 and 4 can be divided into two groups corresponding to fully charged (i.e., protonation probability near 1) or fully uncharged Lys 31 at physiological pH, as indicated for frame 3 in Figure 8. This binary behavior is due to strong interactions with Asp G34 on SecG, which is less than 5 Å away in this frame (measured from the NZ atom of LYS to the CD atom of Asp). This is confirmed by the fact that in the frame 3 structures, Asp G34 shows the same binary titration behavior and protonation state as LYS, meaning when LYS 31 is charged, Asp G34 is charged, too, and when LYS 31 is uncharged, so is Asp G34. The Asp residue is located on the cytosolic loop of SecG at the top of the channel, and is thus free to interact with the peptide in multiple positions. The wide spread of the individual titration curves in other frames also shows the strong dependence of the residue’s protonation probability on its orientation, as the C_α_-atom of the residue is constrained, and so variations are due to thermal fluctuations of the side chain.

**Figure 9:**
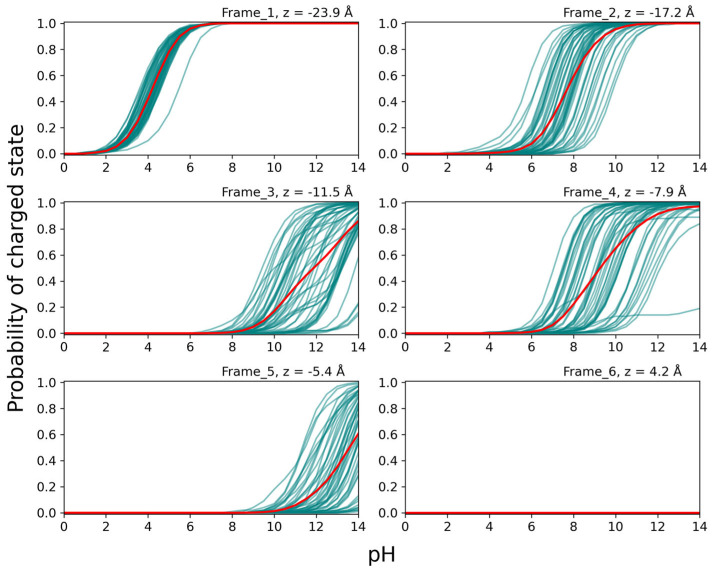
Titration curves of peptide residue GLU 31 over the course of translocation. The curves in frames 1–6 are each based on 50 structures from constrained MD simulations, with the position of residue 31 determined by constant velocity SMD simulations pulling the peptide through the channel. The frames correspond to the positions indicated in Figure 5b. The teal curves are individual titration curves of the 50 structures, while the red lines correspond to the average of the 50 structures in each frame to illustrate the average probability of being negatively charged.

In Figure 9, the titration curves for Glu at position 31 for structures taken from MD simulations with Glu 31 in the GLU protonation form (cf. Figure 7) are shown for the six different positions in the SecYEG channel. For the titration curves of the GLU residue (Figure 9), a general transition to the uncharged state can be seen as the residue travels further down the channel, with the exception of the small increase from frame 3 to 4. Unlike LYS 31, GLU 31 does not return to the charged state once it has passed the PR. This may be due to residues Glu 68 and Glu 195 located at the bottom of the channel—which remain almost entirely charged in frames 4–6—though this was not tested explicitly by fixing their protonation states, as was done for two Asp residues (see below). Additionally, it appears that the protonation state of GLU 31 is less dependent on its orientation, as the individual titration curves have a much smaller spread compared to LYS 31. This observation might be related to the fact that the Glu chain is not only shorter, but also less flexible than that of Lys.

**Figure 10:**
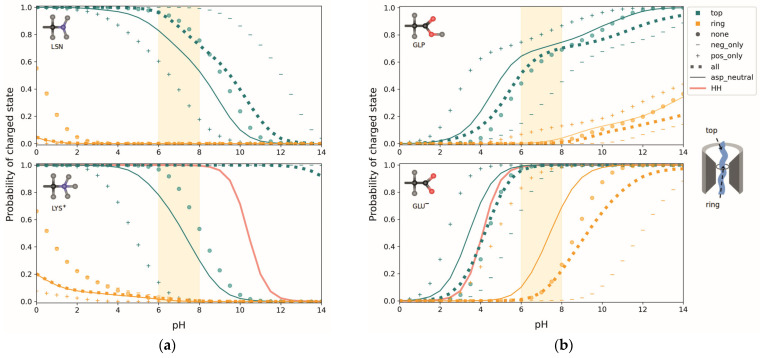
(**a**) Comparison of average titration curves of LSN (top graph) and LYS (bottom graph) at the top of the channel and in the PR. Teal curves show the probability of the residue being in the charged state while at the top of the channel; orange curves when the residue is in the PR. The rectangle marker denotes the unfixed state (“all”), in which all titratable residues are free to titrate, while the solid curves (“asp_neutral”) are with Asp G34 and Asp Y410 fixed in their charge-neutral protonation states (see Supplementary Material SM4, Figures S1 and S2 for graphs with different charge states). The circle marker corresponds to all titratable residues in the protein (except the test residue) being fixed in the uncharged state, while the “+” marker shows all negative residues, and the “−” marker all positive residues fixed in the uncharged state (“none”, “pos_only” and “neg_only” curves, respectively). The salmon curve is the Henderson-Hasselbalch (HH) standard curve for the protonation probability of Lys based on a ($pK_{a}$ of 10.4 [54]. The physiological pH range of 6–8 is highlighted in orange. (**b**) Same as in (**a**), but for GLP (top) and GLU (bottom; see Supplementary Material SM4, Figures S3 and S4 for graphs with different charge states of Asp G34 and Asp Y410). The HH standard curve corresponds to a ($pK_{a}$ of 4.1 [55].

In order to determine the bias in protonation probability introduced by the choice of the protonation form (cf. Section 2.3.5) used for the MD simulation, we performed additional simulations with the alternative protonation forms GLP and LSN. These simulations were done only for two frames in each case referred to as “top” (frame 1, where residue 31 is located in the cytoplasmic entrance in the channel) and “ring” (frame 5 for LSN and frame 4 for GLP, where residue 31 is located in the PR). The average titration curves of the 50 snapshots from the top and ring frames were then compared for the two protonation forms. Additionally, the effect of other titratable residues on the $pK_{a}$ shift in a translocating peptide was investigated by fixing Asp G34 on SecG and Asp Y410 on SecY in different combinations of protonation states and titrating peptide residue 31. To determine the effects of positive and negative charges, one run was done with all Asp and Glu residues (except the test residue) fixed in their uncharged states (“pos_only” curves), and another fixing Arg and Lys residues (except the test residue) in their uncharged states (“neg_only” curves). The net effect of these titratable residues was determined by fixing them all in the uncharged state (“none” curves), so that any $pK_{a}$ shift must be due to the dielectric environment and interaction with non-titrating charges. All titration curve averages are overlaid for comparison in Figure 10.

The averaged titration curves show a largely monotonic behavior, so that an effective $pK_{a}$ value of the titrating group may be defined by the pH for which the probability of the charged state is 0.5. For both LYS and LSN, a shift in the $pK_{a}$ to lower values is apparent in the ring frame compared to the top frame (Figure 10a)—meaning a higher probability for residue 31 being uncharged in the PR at physiological pH. In fact, in this pH range, the probability of Lys being charged in the PR is essentially zero. However, when the deprotonated and uncharged form LSN is used in the MD simulation, the $pK_{a}$ in both the top and the ring frames is lower compared to LYS, indicating a bias towards the uncharged state. Possibly titrations on structures obtained from MD simulations tend to be biased towards the protonation states of the protonation forms chosen.

The largest changes in the top frames is seen when Asp G34 is fixed in the uncharged state, resulting in a $pK_{a}$ shift from >14 to about 7 for the LYS curves (Figure 10a, bottom), while the corresponding shift in the LSN curves is only about 1 pH unit (Figure 10a, top graph). The change in charge state of Asp Y410 does not result in significant shifts. This finding can be explained by the fact that Asp G34 is spatially very close to residue 31 in the top frame. Therefore, there are strong stabilizing interactions between the anionic Asp and cationic Lys, preventing the latter from giving up its proton. As LSN is uncharged during the MD simulation, there are no such interactions and the charge state of Asp G34 has a smaller effect. The influence of Asp G34 on the charge state of Lys 31 is thus dependent on the standard charge states of the protonation forms used in the MD simulations, and is likely exaggerated in the LYS frames. Labeled graphs for the different Asp states can be found in Supplementary Material SM4, Figures S1 and S2.

Switching off the remaining negative charges (“pos_only”) in the top frames lowers the $pK_{a}$ of LYS by another 4 pH units and LSN by about 2, while switching off all positive charges (“neg_only”) has virtually no effect on LYS, but increases the $pK_{a}$ of LSN by 2 pH units. The combined effect of all titratable residues on LSN is negligible, with the “none” and “all” states resulting in nearly identical curves. In the LYS case, the interaction with Asp G34 dominates, resulting in an ∼7 pH unit shift between the “all” and “none” states. Any effects on Lys in the PR are negligible as it is almost entirely uncharged independent of any other titratable residues. What is noticeable, however, is that for both LYS and LSN, the “neg_only” state is identical to the “none” state, showing that negatively charged groups have no significant interactions with positively charged peptide residues when in the PR.

Similar to Lys, Glu experiences a shift towards the uncharged state as it moves through the pore (Figure 10b), as well as a difference in the protonation probabilities between the deprotonated and protonated form used for the MD simulation. Again, the frames using the protonated form (GLP) show a bias towards the protonated state, seen by an increase in $pK_{a}$. Additionally, the GLP residue’s protonation probability seems to be more dependent on its orientation than that of GLU, as is evident from the “double- humped” shape of the average titration curves resulting from a large spread in the curves of individual structures (not shown for GLP). In all cases, though, the “all” and “none” curves are very similar, meaning that the combined effect of titratable residues on the $pK_{a}$ of Glu is very small. The presence of only positive or negative charges would result in a significant shift, as is shown by the curves in the “pos_only” and “neg_only” states. Apparently, the effects of positively and negatively charged groups cancel each other. This is an interesting finding as there are 1.7 times more basic amino acids in SecYEG than acidic ones. The position of Glu in the channel allows for a more balanced interaction with basic and acidic groups, including a strong influence of Asp G34. Labeled graphs for the different Asp states can be found in Supplementary Material SM4, Figures S3 and S4. Overall, the $pK_{a}$ of Glu, like that of Lys, appears to be dictated by the dielectric properties of its environment, interaction with non-titrating charges, and its distance from the dielectric interface—rather than interactions with ionizable residues in the channel.

**Figure 11:**
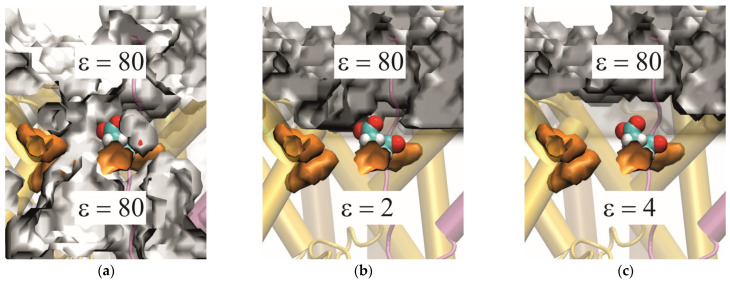
Comparison of (ε(r) interfaces (silver iso-surfaces) of GLU ring frames (position of GLU 31 near the PR as in frame 4 of Figure 5b) for (**a**) an explicit membrane, (**b**) an implicit membrane, and (**c**) an implicit membrane plus dummy atoms. The pore ring is represented by four Ile residues in orange, and the GLU residue is shown in a van der Waals representation. The implicit membrane is set up as a slab of low dielectric permittivity ((ε=2) constrained to certain (z-coordinates (cf. Figure 6). Due to the algorithms in TAPBS, this slab is able to “leak” into the pore and cause an area with (ε=2 inside the channel. (**c**) Placing evenly spaced, uncharged dummy atoms inside the protein blocks the SES probe from entering and allows for assigning (ε=4 to the channel interior.

We have also tested the effect of the precise spatial dependence of $\varepsilon \left(r\right)$, i.e., the position of the interface between high and low dielectric constant in the channel (Figure 11). This was done only for the ring frames, where residue 31 is in the PR. For the first test (Figure 11a), where neither a membrane slab nor dummy atoms, but an explicit membrane was used, the SES probe (sphere of radius 1.4 Å; cf. Section 2.3.4.) was able to travel all the way through the pore—causing the residue to be surrounded by a medium of ε=80. In the test visualized in Figure 11b, there are also no dummy atoms, but an implicit membrane in the form of a slab with ε=2 replacing the explicit lipid bilayer (cf. Figure 6). This resulted in the positioning of a 2/80 interface almost at the position of the test residue. Additionally, due to the algorithms in TAPBS, the low permittivity area of the membrane slab was also defined in the pore region. In both of these setups, the test residue was in contact with the ε=80 interface and thus remained charged. The final panel (Figure 11c) shows the setup generally used in this project: the same implicit membrane as in (b) but with the addition of uncharged dummy atoms in the channel to impede the entry of the SES probe. This procedure results in the entire protein, including the PR area, being assigned ε=4, and holds the ε=80 interface off from protruding almost to the PR. This was the only case in which the test residue changed its protonation state. Thus, if the channel water is modeled as having bulk properties, the peptide residue 31 is not neutralized.

## 4. Discussion

The goal of the present work was to determine the probability of being charged of a test residue in a translocating peptide as it travels through the SecYEG translocon. The simulations clearly suggest that both, Lys and Glu as test residues, are charge-neutralized when they pass the PR, i.e., the constriction in the hydrophobic interior of the translocon. This result argues against a discrimination of positively and negatively charged residues that would suggest the electric component of the PMF directly contributes to the driving force behind peptide translocation in neutralophilic bacteria as previously hypothesized [10]. However, the simulation methods involve a number of approximations that must be discussed, and the results should be confronted with recent experimental studies on the translocation of charged residues [12,56].

PB-based electrostatic methods to determine $pK_{a}$ values are usually applied to X-ray structures of proteins—and probably work best in this context. In the case of active SecYEG, we had neither X-ray nor cryo-EM structures of the translocon in different stages of the translocation process with a sufficiently high resolution at our disposal. So, we decided to create structures of the translocating peptide by employing MD simulations. These simulations were built upon an open *T. thermophilus* structure that was constructed in previous work [5,26] from the closed channel [24] by taking into account information about the opening from an active-state chimera structure [25]. The different stages of translocation were created using an SMD protocol [35] and the test residue fixed at its backbone by a harmonic constraint. One possible way to proceed from there would have been an energy minimization of the respective stages of translocation and PB computations based upon these minimized structures. However, due to the flexibility of the test residue’s side chain, it probably would have been difficult to find a single, representative conformation. Therefore, we decided to perform MD simulations to create an ensemble of structures and to perform PB/MC titrations on each structure. It should be noted that the translocation process is slow (in the range of milliseconds per residue), so that the transported peptide can in fact sample a large number of conformations in each stage of the translocation.

Since the used structures were taken randomly (i.e., with equal weight) from the second half of a 10 ns NPT simulation, the structural ensembles may be considered as representing roughly isothermal-isobaric ensembles. Then, the ensemble-averaged protonation probabilities are obtained by just averaging the individual titration curves as shown in Figure 8 and Figure 9. Clearly, the results may be improved by increasing the number of structures and the length of the NPT trajectories. There is always a compromise between accuracy and simulation time. A problem that cannot be solved within this framework is the bias of the MD simulations due to the explicit protonation form of the titratable residue used in these simulations. Note that any standard MD simulation is limited by this problem. Therefore, it is advisable to perform tests with different protonation forms to assess errors. In the present case, we obtain the same basic result for each protonation form: The probability of the test residue being charged is close to zero when the residue is in the PR.

A critical—and much debated [20,45,57]—aspect of PB computations is the choice of the dielectric constant of the protein. We shall not discuss here the applicability of the macroscopic concepts of medium electrodynamics to the length scale of biomolecules, as this is—to our knowledge—an unsolved fundamental problem. Instead, we work with the widely accepted presumption that PB-based electrostatics provides a meaningful physical model of proteins. The next problem is that ε is actually the real part of a complex function that, for analytical purposes, can be extended to the complex plane [46,58,59]. The value that we use in the computations is the zero-frequency limit, i.e., the static dielectric constant. To understand why it can be used in computations on MD snapshots, one has to note that our MD simulations do not actually serve to study dynamics, but to provide a sampling of structural variations. Each snapshot then corresponds to a static structure, and we are left with two questions: (i) What is the appropriate value of $\varepsilon_{protein}$? And: (ii) What about the water in the channel?

Ad (i): As discussed by Gilson and Honig [45], dipolar groups in a protein play a dual role: They act as sources of the protein’s permanent electrostatic field (represented here by APCs) and also, to the extent that they are free to rotate, contribute to enhance the protein’s dielectric constant over that of electronic polarization ( $\varepsilon_{protein}>\varepsilon_{\infty }$). When we compute on a given snapshot, the APCs represent the permanent electrostatic field in that particular conformation. However, we also have to take into account possible reorientations of polar groups that may take place on the timescale of the equilibration of de/protonation reactions starting from that protein conformation. We think that the moderately high value of $\varepsilon_{protein}=4$ corresponding to protein powders with a low water content [45,46] is suitable for this purpose, particularly for the inner region of a membrane protein.

Ad (ii): The channel in SecYEG, through which the peptide is translocated, contains a lot of water even in the presence of the peptide. Clearly, for a PB/MC computation, the explicit water has to be removed to avoid biasing of the MC sampling; the water must be represented implicitly by a dielectric continuum, and the question arises as to what value of ε is appropriate here. The algorithms in TABPS assign to each cavity, which is able to accommodate a sphere of radius 1.4 Å (corresponding to a water molecule) a value of $\varepsilon_{aq}=80$. Such an assignment is also made to a significant part of the SecYEG channel so that the test residue 31 still is in contact with a highly polarizable environment when being positioned at the PR (Figure 11a). From a molecular point of view, the high dielectric constant means that the water molecules in this region are free to rotate, so that they basically behave like bulk water. However, it is questionable that water in the channel behaves like that.

Earlier numerical simulations suggest that confined water should have a significantly smaller dielectric constant than bulk water [60,61,62]. This prediction was confirmed experimentally by Fumagalli et al. [63] for liquid water within nanochannels. Seyedi and Matyushov [64] computed the dielectric constant of the hydration shell of cytochrome *c* based on MD simulations and found a value of ε≈4 at room temperature. Therefore, we think that assigning such a low dielectric constant to the whole interior of SecYEG, including the water-filled regions, is physically realistic. This assumption is crucial, as we found that only with such a low dielectric constant, is it possible to achieve charge neutralization of the test residue. So, we conclude that the low dipolar susceptibility of water confined in the channel during the translocation process critically contributes to the preference of the titratable residues Lys and Glu being transported in the uncharged state. This behavior simply reflects the energetic cost of transferring a charge from an aqueous environment to a less polar one, which according to the approximation provided by Born’s formula for ions

(18) $$\Delta G_{transfer}=-\frac{N_{A}q^{2}}{8\pi \varepsilon_{0}r_{0}}\left(\frac{1}{\varepsilon_{aq}}-\frac{1}{\varepsilon_{protein}}\right),$$

where $r_{0}$ is the radius of the ion, does not depend on the sign of the charge q. The same is true for the more elaborate computations based on the LPBE. Thus, if the dielectric properties of the interior of SecYEG determine the protonation state changes, no discrimination between acids and bases is to be expected. The only exception could be arginine (Arg) due to its high reference $pK_{a}$ (see below).

**Figure 12:**
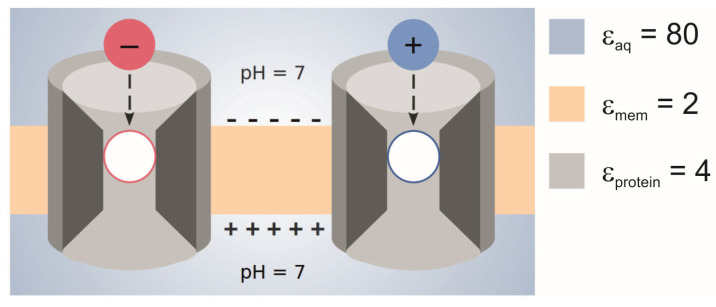
Visual summary of the titration behavior of acids and bases as determined in this work. The blue and red circles represent the charged states of acidic and basic amino acids, while the white circles show the uncharged states. The magnitude of the dielectric constants used in the calculations, as well as the areas in which they were defined, are shown in the legend.

Previous MD simulations [65], performed on the basis of a SecYE structure from the hyperthermopile archaeon *Pyrococcus furiosus* [66], revealed an anomalous behavior of water inside the channel. The rotational relaxation time of water molecules (defined as the time required for the dipole autocorrelation function to decay to 1/e), which is 1 ps in bulk water, was found to increase to 20–30 ps inside SecY and even above the observation time near the center of SecY. It should be noted that the structure used for these simulations is highly distorted and it is unclear which state of the translocon it represents. Nonetheless, the simulations can be taken as a hint that the rotational dynamics of channel-bound water is retarded, which may be related to a decreased dielectric constant. Another important result from these simulations is that the water molecules are highly oriented inside SecYE. This finding, together with the retarded reorientation, hints at a problem: Oriented water molecules can stabilize the charged state of a titratable residue by virtue of their dipole. Such behavior is not covered by a dielectric continuum model with a low dielectric constant. Rather, one would have to put explicit water molecules with non-zero APCs in the model. However, explicit water molecules are prohibitive in PB/MC titrations as they bias the MC sampling as mentioned above. A solution to this problem would be a constant-pH MD simulation [67] employing a non-equilibrium MD/MC approach [68] or λ-dynamics [69]. However, these methods are expensive, are outside the scope of the present project, and have their own problems [67]. Nevertheless, our conclusion that Lys and Glu are charge-neutralized during translocation (Figure 12) has to be drawn subject to the proviso that our continuum model mimics the behavior of channel water well enough.

In their extensive study on the rate-limiting transport of positively charged residues through the Sec-machinery, Allen et al. [56] showed that translocation is retarded by Arg residues in the peptide, while no such retardation is observed for Lys. Allen et al. explain the different behavior of Lys by a deprotonation, which is principally in agreement with our findings. To substantiate their proposal, Allen et al. [56] performed an in silico $pK_{a}$ analysis based on MD snapshots (by successively substituting each peptide residue with Lys), but they employed the PROPKA3.1 program [70]. While the $pK_{a}$ computation in PROPKA is structure-based, it is rather empirical than physics-based. In particular, the long-range electrostatic interaction of a titratable group with background charges is computed only within a radius of 6 Å, and coupled sites are not treated with MC, but by employing empirical rules [70,71]. Also, it remains unclear how the channel water is included in the computations performed by Allen et al. [56]. Another problem is that for each position in the channel, a different peptide was investigated. Remarkably, though, Allen et al. found a $pK_{a}$ decrease for Lys inside the channel down to about 6.5. While this $pK_{a}$ shift is probably not sufficient to ensure deprotonation at physiological pH, it qualitatively points in the same direction as our simulations.

We do not know how Arg at position 31 in the peptide would behave under our simulation conditions. On one hand, it has a high reference $pK_{a}$ value of 13.8 [72], and Arg is generally considered to stay protonated even when buried inside the protein [73]. On the other hand, it could be deprotonated if it experiences a $pK_{a}$ shift in the same order of magnitude as Lys in our simulations. In our opinion, the significance of positively charged residues in a translocating peptide for barriers to transport is not clear. Allen et al. [56] found a major barrier in the free energy profile for translocation (based on the coarse-grained MARTINI 2.2 force field [74,75]) that is dominated by the positively charged Lys residue at the PR and significantly reduced by de-charging the Lys. In our previous simulation of the Lys-peptide [5] (based on the all-atom CHARMM36m force field), we found several barriers that are rather determined by bulky amino acids than a single charged residue—although we cannot exclude that de-charging Lys would alter the free energy profile. We believe that the role of net charges for the translocation barrier is overestimated. The different behavior of Arg compared to Lys could also result from the bulkiness of the guanidino group. Further simulations are necessary to clarify this point.

In line with this argumentation is the finding of Allen et al. [56] that the stimulation of translocation by the PMF does not significantly depend on the (presumed) net charge of the peptide. On the other hand, there is a stimulation of translocation by the PMF. We have shown in our previous work [5] that the transmembrane potential, which is part of the PMF, affects the orientation of transmembrane α-helices and, in this way, can contribute to an opening of the channel. Thus, the effect of the PMF is more complicated than simply dragging negatively charged residues through the PR, meaning that it is not required that a Glu residue in the peptide remains charged.

Ismail et al. [12] found evidence of a force acting on a translocating peptide containing an Asp_5_ segment (5D stretch), which they ascribed to the transmembrane potential—based on experiments with the uncoupler indole. According to our understanding of these results, the force is exerted on the 5D stretch while it is located in the cytoplasmic vestibule of the translocon. Based on our simulations, we speculate that the 5D stretch is still highly charged in this position, although not necessarily fully charged because of the mutual electrostatic interaction of the five Asp residues. It remains to be clarified, how these charges—in conjunction with the transmembrane potential—facilitate translocation of the 5D stretch.

In the present work, we concentrated on the course of the translocating residue from the cytoplasmic vestibule of SecYEG to the PR. However, some frames also cover the periplasmic region below the PR (Figure 5, Figure 8, and Figure 9). Here, we made the interesting observation that Lys and Glu behave differently: Lys is recharged, while Glu stays uncharged. In the light of a simple model of how the transmembrane potential acts on charged residues, this finding is counterintuitive, since a positive charge at the periplasmic side is not favored. The focus of our work was on the behavior of the charged groups when they enter the PR region; we investigated only one frame, in which the residue has passed the PR (frame 8 for Lys and frame 6 for Glu, cf. Figure 5). More simulations are required before the titration behavior of the peptide at the periplasmic side of the membrane and the role of the PMF in the later stages of the translocation can be adequately discussed. Those investigations should also consider the charge state of Asp 23, which is located next to the cleavage site in the peptide (Figure 4a) and could influence the translocation process by interacting with the transmembrane potential in the periplasmic region.

It remains a challenge to directly include the protein structural flexibility in $pK_{a}$ calculations. Our work has confirmed that fixing the protonation form during an MD simulation causes a bias. Therefore, protonation state changes should be accounted for during the simulation of the dynamics. This goal can be achieved with the constant-pH MD methods mentioned above [67,68,69], which could also solve the problem of explicit water.

## 5. Conclusions

Based on calculations performed on a titratable residue in a peptide during translocation in SecYEG, it appears that the farther into the channel a residue travels, the less likely it is to be found in the charged state. This was shown for Lys and Glu. Thus, the charge neutralization is independent of the sign of the charged state. The channel does not stabilize one charge over the other, which is expected as the low dielectric permittivity within the channel plays a decisive role. We expect Asp residues to behave like Glu under these conditions, but we cannot say yet whether Arg would be charge-neutralized too. It was also shown that the protonation form of the residue used in the MD simulation biases the protonation probability significantly, although this artifact had no influence on our basic result. This result is visualized in Figure 12 with reference to the hypothesis depicted in Figure 2. Since there is no discrimination between acids and bases regarding charge neutralization, we conclude that the transmembrane potential plays no direct role in pulling negatively charged residues through the PR, while positively charged residues need to be deprotonated. However, we do not exclude the possibility that the transmembrane potential exerts a force on a charged residue while being located in the cytoplasmic vestibule of the translocon. Details of how the PMF facilitates translocation under these conditions and the role it plays at the periplasmic side remain to be clarified. Note that we cannot yet comment on the behavior of the charged groups at the periplasmic side of the membrane, and as indicated by “pH = 7” in Figure 12, we only investigated the case of neutralophiles and did not consider ΔpH effects in our simulations. These problems are left for future work.

The calculations done in this project are based on continuum electrostatics models and thus rely on the assumptions that (i) the interior of the protein, including the channel water, has a low dielectric permittivity, and (ii) stabilizing effects of oriented water molecules/hydration shells around the residues are not strong enough to compensate for the more hydrophobic environment. The complexity of the SecY channel—containing confined water with distinctly non-bulk properties in its pore—warrants the use of more elaborate models, and constant-pH MD simulations are a possible next step in investigating the titration behavior of translocating peptides.

## Data Availability

The original contributions presented in this study are included in the article/Supplementary Material. Further inquiries can be directed to the corresponding author.

## Supplementary Materials

The following supporting information can be downloaded at https://www.mdpi.com/article/10.3390/biom15101442/s1: SM1: PQR parameters for non-standard molecules; SM2: State description of GLP; SM3: State description of LSN; SM4: Titration curves (Figures S1–S4).
