# Supplementary material for: Charge Neutralization During Peptide Transport in the Bacterial SecYEG Translocon
Source: Biomolecules. 2025 Oct 12;15(10):1442. doi: 10.3390/biom15101442 (PMC12562832; doi:10.3390/biom15101442)
Supplement: Supplementary file 1 [file biomolecules-15-01442-s001.zip › biomolecules-3807917-supplementary.pdf]

Supplementary Material

Charge Neutralization during Peptide Transport in the Bacterial SecYEG Translocon

Laura Nübl, Ekaterina Sobakinskaya and Frank Müh*

Johannes Kepler University Linz, Institute for Theoretical Physics, Altenberger Strasse 69, 4040 Linz, Austria
* E-mail: frank.mueh@jku.at

Contents

SM1: PQR Parameters for Non-standard Molecules 2

SM2: State Description File for GLP 3

SM3: State Description File for LSN 3

SM4: Titration Curves 4

Figure S1 4
Figure S2 5
Figure S3 6
Figure S4 7

SM1: PQR Parameters for Non-standard Molecules

RESI	GLP	0.00		
ATOM	N	NH1	−0.4700	1.8500
ATOM	HN	H	0.3100	0.2245
ATOM	CA	CT1	0.0700	2.0000
ATOM	HA	HB1	0.0900	1.3200
ATOM	CB	CT2	−0.1800	2.0100
ATOM	HB1	HA2	0.0900	1.3400
ATOM	HB2	HA2	0.0900	1.3400
ATOM	CG	CT2	−0.2100	2.0100
ATOM	HG1	HA2	0.0900	1.3400
ATOM	HG2	HA2	0.0900	1.3400
ATOM	CD	CC	0.7500	2.0000
ATOM	OE1	OB	−0.5500	1.7000
ATOM	OE2	OH1	−0.6100	1.7000
ATOM	HE2	H	0.4400	0.2245
ATOM	C	C	0.5100	2.0000
ATOM	O	O	−0.5100	1.7000

RESI	LSN	0.00		
ATOM	N	NH1	−0.4700	1.8500
ATOM	HN	H	0.3100	0.2245
ATOM	CA	CT1	0.0700	2.0000
ATOM	HA	HB1	0.0900	1.3200
ATOM	CB	CT2	−0.1800	2.0100
ATOM	HB1	HA2	0.0900	1.3400
ATOM	HB2	HA2	0.0900	1.3400
ATOM	CG	CT2	−0.1800	2.0100
ATOM	HG1	HA2	0.0900	1.3400
ATOM	HG2	HA2	0.0900	1.3400
ATOM	CD	CT2	−0.1800	2.0100
ATOM	HD1	HA2	0.0900	1.3400
ATOM	HD2	HA2	0.0900	1.3400
ATOM	CE	CT2	0.1300	2.0100
ATOM	HE1	HA2	0.0750	1.3400
ATOM	HE2	HA2	0.0750	1.3400
ATOM	NZ	NH3	−0.9600	1.8500
ATOM	HZ1	HC	0.3400	0.2245
ATOM	HZ2	HC	0.3400	0.2245
ATOM	C	C	0.5100	2.0000
ATOM	O	O	−0.5100	1.7000

RESI	DUM	0.00		
ATOM	DUM	DUM	0.000	2.000

RESI	TIP3	0.00		
ATOM	OH2	OT	0.0000	1.7682
ATOM	H1	HT	0.0000	0.2245
ATOM	H2	HT	0.0000	0.2245

SM2: State Description File for GLP

```
0.00  pK  R
ATOM 12754    CG     GLU A 815       9999.9999999.9999999.999-0.21099.999       PB9C C
ATOM 12757    CD     GLU A 815       9999.9999999.9999999.999 0.75099.999       PB9C C
ATOM 12758    OE1    GLU A 815       9999.9999999.9999999.999-0.55099.999       PB9C O
ATOM 12759    OE2    GLU A 815       9999.9999999.9999999.999-0.61099.999       PB9C O
ATOM 12760    HE2    GLU A 815       9999.9999999.9999999.999 0.44099.999       PB9C H
4.40  pK  D
ATOM 12754    CG     GLU A 815       9999.9999999.9999999.999-0.28099.999       PB9C C
ATOM 12757    CD     GLU A 815       9999.9999999.9999999.999 0.62099.999       PB9C C
ATOM 12758    OE1    GLU A 815       9999.9999999.9999999.999-0.76099.999       PB9C O
ATOM 12759    OE2    GLU A 815       9999.9999999.9999999.999-0.76099.999       PB9C O
ATOM 12760    HE2    GLU A 815       9999.9999999.9999999.999 0.00099.999       PB9C H
```

SM3: State Description File for GLP

```
0.00  pK  R
ATOM 12812    CE     LYS A 818       9999.9999999.9999999.999 0.13099.999       PB9C C
ATOM 12813    HE1    LYS A 818       9999.9999999.9999999.999 0.07599.999       PB9C H
ATOM 12814    HE2    LYS A 818       9999.9999999.9999999.999 0.07599.999       PB9C H
ATOM 12815    NZ     LYS A 818       9999.9999999.9999999.999-0.96099.999       PB9C N
ATOM 12816    HZ1    LYS A 818       9999.9999999.9999999.999 0.34099.999       PB9C H
ATOM 12817    HZ2    LYS A 818       9999.9999999.9999999.999 0.34099.999       PB9C H
–10.40  pK  P
ATOM 12812    CE     LYS A 818       9999.9999999.9999999.999 0.21099.999       PB9C C
ATOM 12813    HE1    LYS A 818       9999.9999999.9999999.999 0.00599.999       PB9C H
ATOM 12814    HE2    LYS A 818       9999.9999999.9999999.999 0.00599.999       PB9C H
ATOM 12815    NZ     LYS A 818       9999.9999999.9999999.999-0.29099.999       PB9C N
ATOM 12816    HZ1    LYS A 818       9999.9999999.9999999.999 0.49099.999       PB9C H
ATOM 12817    HZ2    LYS A 818       9999.9999999.9999999.999 0.49099.999       PB9C H
```

SM4: Titration Curves

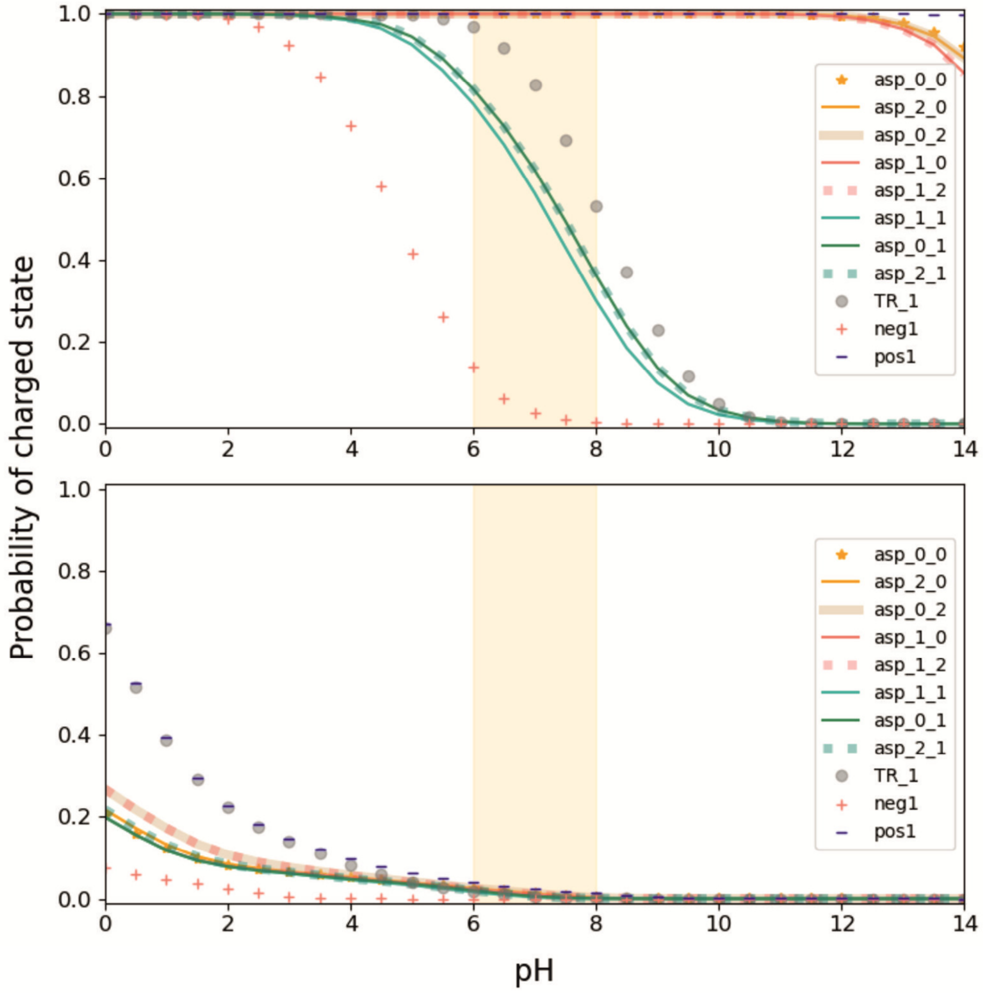

Figure S1. Overlay of average titration curves of lysine (LYS) at position 31 of the peptide located in the top frame (top) or the ring frame (bottom; cf. Figure 5a of the main text), showing the effects of the protonation states of titratable residues in the translocon on its pK_a. The legend shows the different combinations of charge states of two specific aspartates with a naming scheme of "asp"_"*state_Asp Y410*"_"*state_Asp G34*", referring to residues 410 in SecY, and 34 in SecG. The number 0, 1, and 2 indicate "unfixed", "fixed in neutral state", and "fixed in charge state", respectively. Thus, the curve "asp_0_0" corresponds to the unfixed state. In "TR_1", "pos1", and "neg1", all, only positive, and only negatively charged titratable residues were fixed in the uncharged state, respectively. The orange bar highlights the physiological pH range.

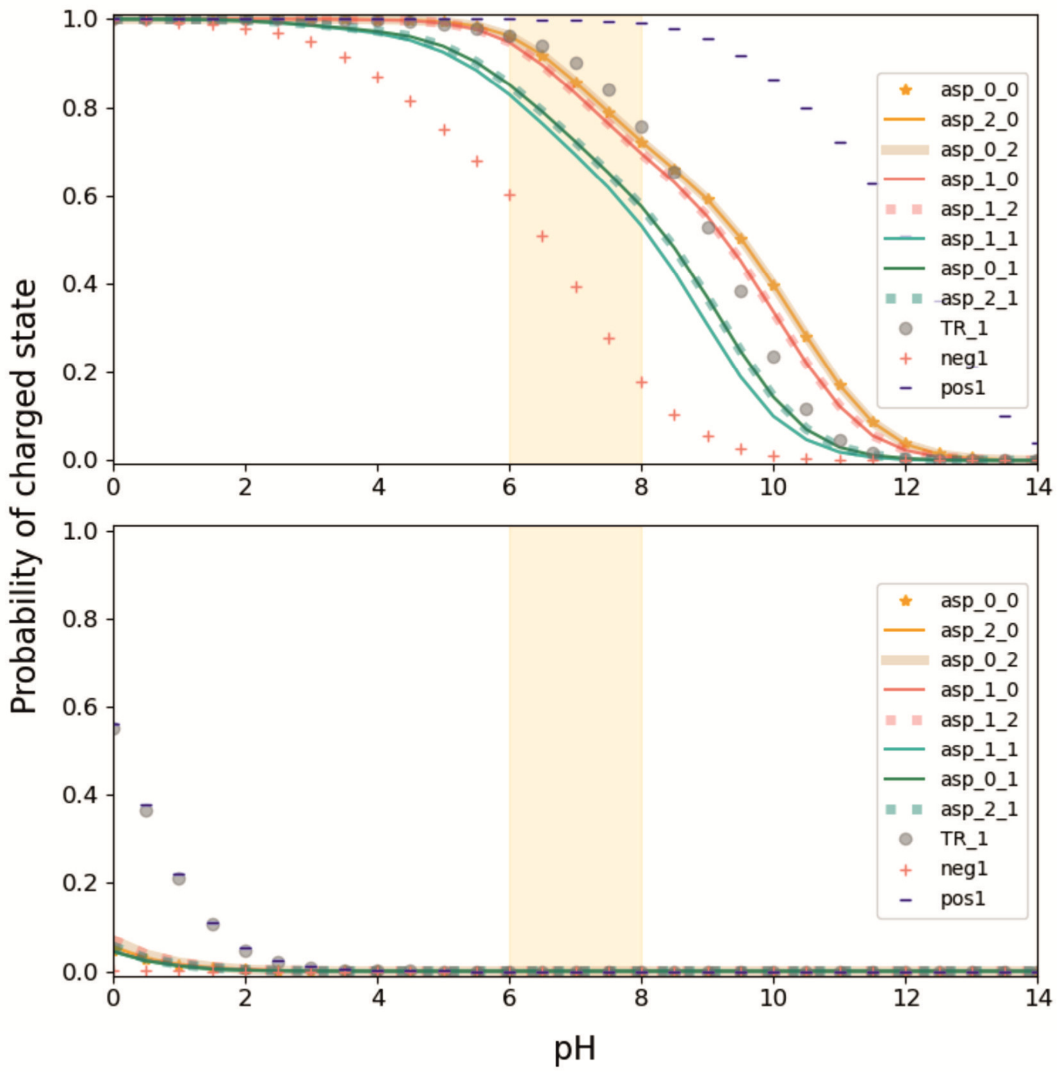

Figure S2. Overlay of average titration curves of LSN 31 of the peptide located in the top frame (top) or the ring frame (bottom; cf. Figure 5a of the main text), showing the effects of the protonation states of titratable residues in the translocon on its pK_a. The legend is explained in the caption of Figure S1.

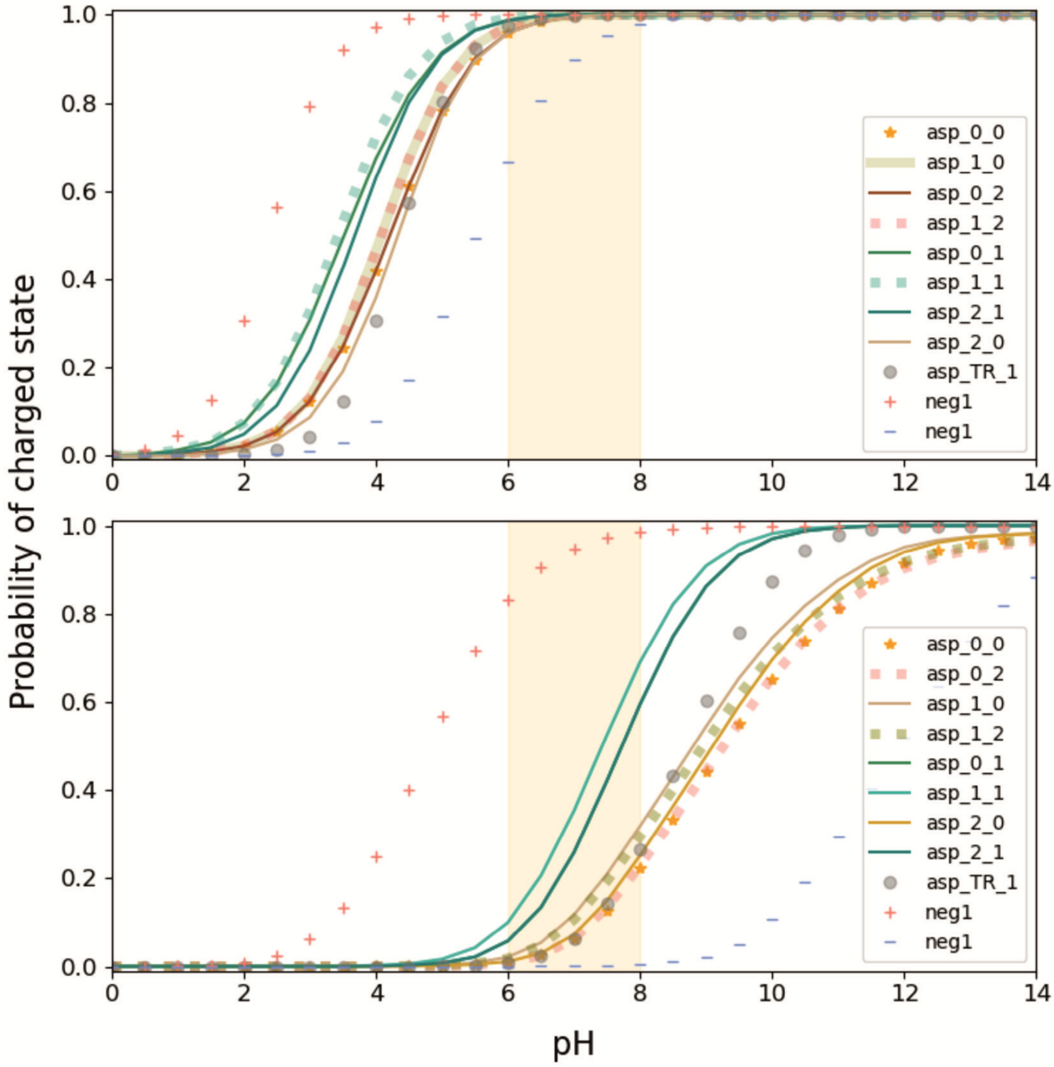

Figure S3. Overlay of average titration curves of GLU 31 of the peptide located in the top frame (top) or the ring frame (bottom; cf. Figure 5b of the main text), showing the effects of the protonation states of titratable residues in the translocon on its pK_a. The legend is explained in the caption of Figure S1.

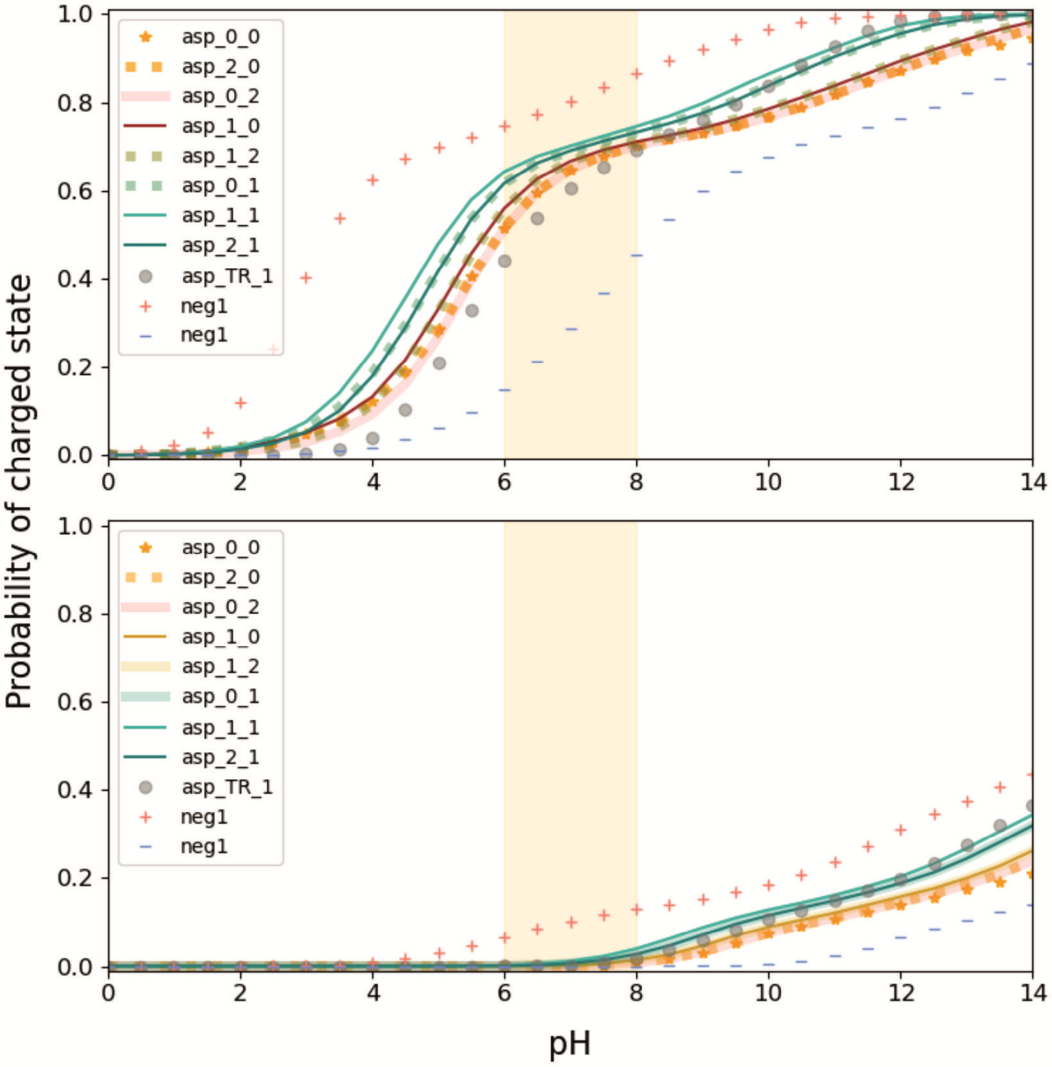

Figure S4. Overlay of average titration curves of GLP 31 of the peptide located in the top frame (top) or the ring frame (bottom; cf. Figure 5b of the main text), showing the effects of the protonation states of titratable residues in the translocon on its pK_a. The legend is explained in the caption of Figure S1.
